# Effect of Atorvastatin on Serial Changes in Coronary Physiology and Plaque Parameters

**DOI:** 10.1016/j.jacasi.2022.07.010

**Published:** 2022-11-01

**Authors:** Cheol Hyun Lee, Jongmin Hwang, In-Cheol Kim, Yun-Kyeong Cho, Hyoung-Seob Park, Hyuck-Jun Yoon, Hyungseop Kim, Seongwook Han, Seung-Ho Hur, Kwon-Bae Kim, Jin Young Kim, Jin-Wook Chung, Joo Myung Lee, Joon-Hyung Doh, Eun-Seok Shin, Bon-Kwon Koo, Chang-Wook Nam

**Affiliations:** a Division of Cardiology, Department of Internal Medicine, Keimyung University Dongsan Hospital, Daegu, Republic of Korea; b Department of Radiology, Keimyung University Dongsan Hospital, Daegu, Republic of Korea; c Division of Cardiology, Department of Internal Medicine, Keimyung University Daegu Dongsan Hospital, Daegu, Republic of Korea; d Division of Cardiology, Department of Internal Medicine, Heart Vascular Stroke Institute, Samsung Medical Center, Sungkyunkwan University School of Medicine, Seoul, Republic of Korea; e Department of Medicine, Inje University Ilsan Paik Hospital, Goyang, Republic of Korea; f Department of Internal Medicine, Ulsan University Hospital, Ulsan, Republic of Korea; g Department of Internal Medicine and Cardiovascular Center, Seoul National University Hospital, Seoul, Republic of Korea

**Keywords:** fractional flow reserve, intermediate coronary artery disease, statin therapy, CAD, coronary artery disease, CFR, coronary flow reserve, FFR, fractional flow reserve, IMR, index of microcirculatory resistance, IVUS, intravascular ultrasound, LDL-C, low-density lipoprotein cholesterol, LLT, lipid-lowering therapy, MLA, minimal lumen area, OR, odds ratio, Pa, proximal aortic pressure, PAV, percent atheroma volume, Pd, distal coronary pressure, TAV, total atheroma volume, T_mn_, mean transit time, VH, virtual histology

## Abstract

**Background:**

The effects of statin on coronary physiology have not been well evaluated.

**Objectives:**

The authors performed this prospective study to investigate changes in coronary flow indexes and plaque parameters, and their associations with atorvastatin therapy in patients with coronary artery disease (CAD).

**Methods:**

Ninety-five patients with intermediate CAD who received atorvastatin therapy underwent comprehensive physiological assessments with fractional flow reserve (FFR), coronary flow reserve, index of microcirculatory resistance, and intravascular ultrasound at the index procedure, and underwent the same evaluations at 12-month follow-up. Optimal low-density lipoprotein cholesterol (LDL-C) was defined as LDL-C <70 mg/dL or ≥50% reduction from the baseline. The primary endpoint was a change in the FFR.

**Results:**

Baseline FFR, minimal lumen area, and percent atheroma volume (PAV) were 0.88 ± 0.05, 3.87 ± 1.28, 55.92 ± 7.30, respectively. During 12 months, the percent change in LDL-C was -33.2%, whereas FFR was unchanged (0.87 ± 0.06 at 12 months; *P* = 0.694). Vessel area, lumen area, and PAV were significantly decreased (all *P* values <0.05). The achieved LDL-C level and the change of PAV showed significant inverse correlations with the change in FFR. In patients with optimally modified LDL-C, the FFR had increased (0.87 ± 0.06 vs 0.89 ± 0.07; *P* = 0.014) and the PAV decreased (56.81 ± 6.44% vs 55.18 ± 8.19%; *P* = 0.031), whereas in all other patients, the FFR had decreased (0.88 ± 0.05 vs 0.86 ± 0.06; *P* = 0.025) and the PAV remained unchanged.

**Conclusions:**

In patients with CAD, atorvastatin did not change FFR despite a decrease in the PAV. However, in patients who achieved the optimal LDL-C target level with atorvastatin, the FFR had significantly increased with decrease of the PAV. (Effect of Atorvastatin on Fractional Flow Reserve in Coronary Artery Disease [FORTE]; NCT01946815)

Statins play major roles in treating patients with atherosclerotic cardiovascular disease by preventing the progression and stabilization of atherosclerosis.^1^, ^2^, ^3^, ^4^ The current guidelines recommend to achieve optimal low-density lipoprotein cholesterol (LDL-C) with a maximally tolerated statin-based intensive lipid-lowering therapy (LLT) in these patients.^5^^,^^6^ The strong recommendations in the guidelines are supported by evidence from large randomized trials and meta-analyses that have a consistent relationship in reducing major adverse cardiovascular events.^2^^,^^7^^,^^8^ In another aspect, several studies using serial intravascular imaging have also demonstrated the beneficial effects of statin on coronary atherosclerosis, which were summarized as stabilizing the plaque with a negative remodeling effect on the vessel, mainly through regression of plaque volume.^3^^,^^9^, ^10^, ^11^ However, the effects of statin on coronary physiology have rarely been evaluated in patients with coronary artery disease (CAD), and the comparative effects of statin therapy on changes in coronary physiology and plaque morphology also have not been established yet. Therefore, the current study aimed to investigate the changes in coronary physiological indices after atorvastatin therapy and their associations with plaque parameters in patients with CAD.

## Methods

### Study design

**Figure 1:**
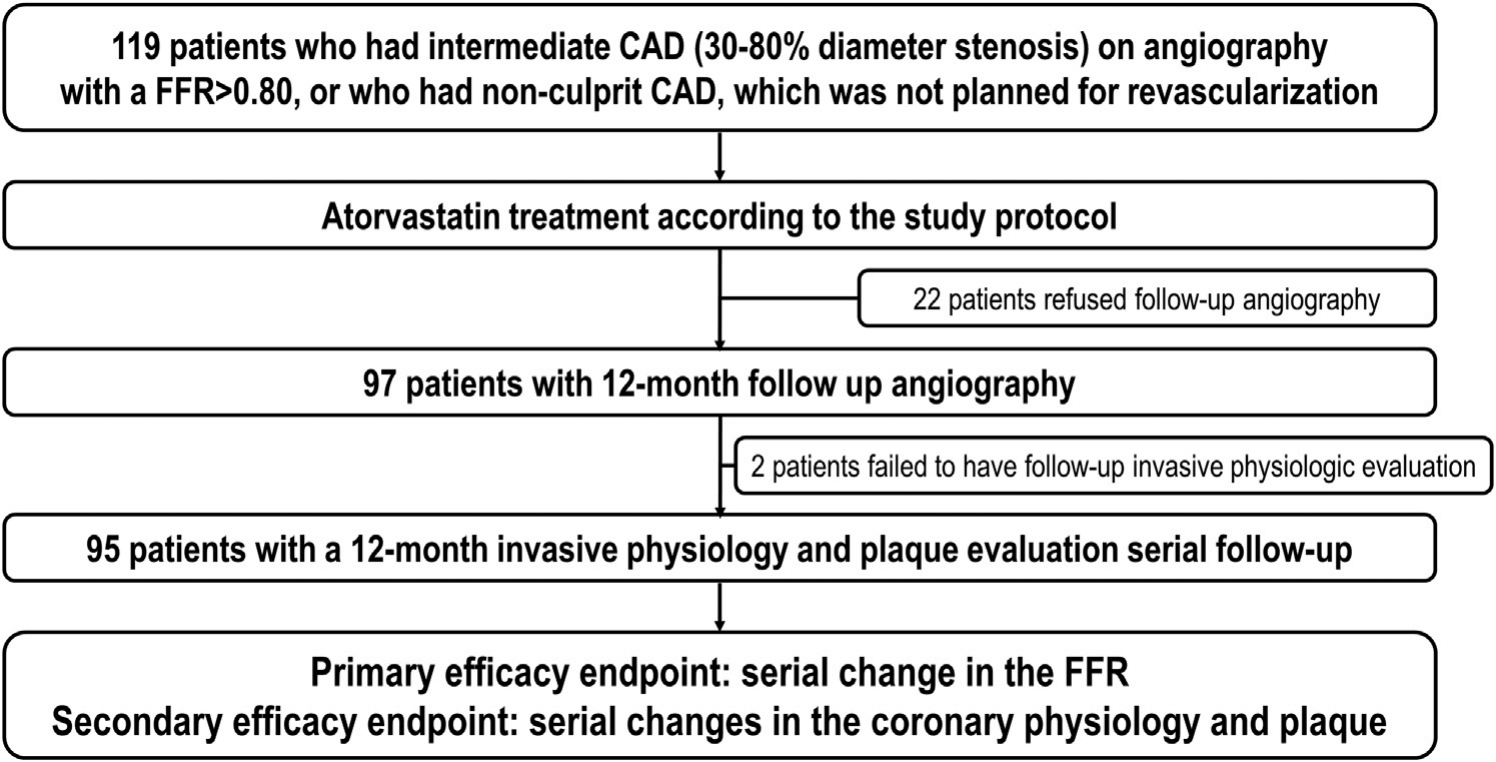
Study Flow Diagram This prospective study investigates the changes in coronary flow indexes and plaque parameters, and their associations with atorvastatin therapy in patients with coronary artery disease (CAD). FFR = fractional flow reserve.

The FORTE (Effect of atorvastatin on Fractional flOw reserve in coronary aRTEry disease) trial was a prospective, open-label, multicenter trial. Patients at least 18 years of age, who had intermediate CAD (30%-80% diameter stenosis by visual estimation) with a fractional flow reserve (FFR) of >0.80 or who had nonculprit lesions not planned for revascularization (Figure 1), were enrolled from 4 South Korean university hospitals (Inje University Ilsan Paik Hospital, Keimyung University Dongsan Hospital, Ulsan University Hospital, and Seoul National University Hospital). The inclusion and exclusion criteria for patient enrollment are listed in Supplemental Table 1. After the diagnostic angiography, invasive physiological assessments for the target vessel and intravascular ultrasound (IVUS) were performed. All coronary physiological and imaging measurements were performed in an independent core laboratory.

During the follow-up, the recommended target goal with atorvastatin therapy was LDL-C <70 mg/dL or ≥50% LDL-C reduction compared with baseline.^12^^,^^13^ The patients who achieved LDL-C target goal were defined as the optimal treatment group, and those who did not achieve the target goal as the suboptimal treatment group. Follow-up coronary angiography, FFR, coronary flow reserve (CFR) and index of microcirculatory resistance (IMR) measurements, and IVUS were performed 12 months after the index procedure. Clinical follow-up and drug compliance were assessed at each visit after enrollment.

The primary efficacy parameter was the change in FFR between baseline and 12-month follow-up. Secondary efficacy parameters were any changes in IVUS measurements, CFR, IMR, and any major cardiac adverse events, which were defined as a composite of death from any cause, any myocardial infarction, or target vessel repeat revascularization. The safety endpoint included the incidence of any adverse reactions caused by the study drug and the incidence of drug discontinuation. The institutional review boards of all participating centers approved the study protocol (NCT01946815), which was in accordance with the Declaration of Helsinki. All patients provided written informed consent.

### Coronary physiological measurements

A 5- to 7-F guiding catheter without side holes was used to engage the coronary artery, and a pressure-temperature sensor-tipped guidewire was used with a 0.014-inch pressure guidewire (St Jude Medical) to measure the pressure. The FFR was calculated as the ratio between the mean distal coronary pressure (Pd) and mean proximal aortic pressure (Pa) at maximal hyperemia. Hyperemia was induced with an intravenous continuous infusion of adenosine (140 μg/kg/min). The pressure sensor was positioned at the distal segment of a target vessel, and intracoronary nitrate (200 mg) was administered before each measurement. To derive the resting mean transit time (T_mn_), a thermodilution curve was obtained by using 3 injections of 4 mL of room temperature saline, and hyperemic Pa, Pd, and T_mn_ were measured during sustained hyperemia. The CFR was calculated as the ratio of resting T_mn_/hyperemic T_mn_. The IMR was calculated using Pd × T_mn_ during hyperemia. The evaluation of FFR was measured that the sensor of the FFR wire was placed the distal 1/3 of the target vessel or at least 20 mm below the target lesion. In addition, in follow-up FFR evaluation, fluoroscopic image capture was used as a reference during the index procedure to match the FFR measured position between the index procedure and follow-up.

### QCA and IVUS measurements

All coronary angiograms were analyzed using standard definitions and measurements in a quantitative coronary angiography (QCA) core laboratory using a dedicated software (Quantcor QCA, Pie Medical). The measured angiographic variables were the reference diameter, the minimal lumen diameter, and the percent diameter stenosis.

Standard IVUS imaging was performed using an automated motorized pullback system (0.5 mm/s; Volcano Corporation). The minimal lumen area (MLA) was obtained at the site of the smallest lumen. To standardize the vessel size, the percent atheroma volume (PAV) (defined as the atheroma volume divided by the vessel volume) and normalized total atheroma volume (TAV) (defined as the summation of the atheroma volume divided by the lesion length) were calculated using volumetric analysis.^10^ The remodeling index was calculated as the ratio of the vessel area at the MLA site/the average of the proximal and distal reference segment vessel areas. Off-line IVUS analyses of all imaged segments and IVUS-virtual histology (VH), plaque components were categorized as fibrous tissue, fibrofatty plaque, necrotic core, or dense calcium and reported as percentages of total plaque areas and volumes were performed at an independent IVUS core laboratory at Keimyung University Dongsan Hospital by an experienced operator blinded to the QCA and FFR values.

### Statistical analysis

Data are reported as frequencies and percentages for dichotomous and categorical variables and as the mean ± SD for continuous variables. Dichotomous and categorical variables were assessed using the chi-square test and Fisher exact test, and continuous variables were assessed using the independent sample *t* test or the Mann-Whitney *U* test, as appropriate. Laboratory, physiological, and IVUS parameters were compared using the paired sample *t* test or the Signed Wilcoxon rank-sum test. Correlations between changes in FFR and PAV were evaluated using Pearson correlation analysis. Independent sample *t* test was used to analyze the difference of the change of FFR, PAV, CFR, and IMR according to the group of the response to atorvastatin or dose of atorvastatin therapy. Independent predictors of a decrease of FFR after a 12-month follow-up were entered into a multivariable logistic regression analysis including parameters in Table 1 for model 1 and the added change of parameters in Table 2 for model 2 except Pd/Pa. For the sample size calculation, the FFR in patients with angiographically intermediate or nonculprit coronary lesions was assumed to be 0.87 ± 0.06 based on the results of our previous study.^14^ It was estimated that atorvastatin therapy would increase the FFR by 0.02 after 12 months. With the power of 90% and the level of significance of 0.05, 95 patients were needed. With an estimated drop-out rate of 20%, 119 patients were, thus, required for the analysis. All analyses were performed using SPSS software version 22.0 (SPSS Inc.) and the R programming language.

**Table 1:** Baseline Clinical and Angiographic Characteristics of the Patients (N = 95)

Age, y	60.5 ± 8.9
Men	73 (76.8)
Body mass index, kg/m^2^	25.4 ± 3.2
Hypertension	47 (49.5)
Diabetes mellitus	18 (18.9)
Hyperlipidemia	27 (28.4)
Current smoker	27 (28.4)
Previous CVA	7 (7.4)
Previous PCI	4 (4.2)
Clinical presentation	
Stable angina	42 (44.2)
Acute coronary syndrome	53 (55.8)
Intensity of atorvastatin	
20 mg	48 (50.5)
40 mg	32 (33.7)
80 mg	15 (15.8)
Discharge medications	
Aspirin	89 (93.7)
ADP receptor antagonist	90 (94.7)
Beta-blocker	48 (50.5)
Calcium-channel blocker	25 (26.3)
ACE inhibitor or ARB	31 (32.6)
Angiographic analysis	
Multi-vessel disease	71 (74.7)
Target lesion	
Left anterior descending artery	41 (43.2)
Left circumflex artery	20 (21.1)
Right coronary artery	34 (35.8)
Reference vessel diameter, mm	3.2 ± 0.5
Minimal lumen diameter, mm	1.5 ± 0.4
Percent diameter stenosis, %	52.6 ± 8.3
Lesion length, mm	19.1 ± 6.9

Values are mean ± SD or n (%).

ACE = angiotensin-converting enzyme; ADP = adenosine diphosphate; ARB = angiotensin II receptor blocker; CVA = cerebrovascular accident; PCI = percutaneous coronary intervention.

**Table 2:** Laboratory, Physiological, and Intravascular Imaging Results of the Enrolled Patients After Atorvastatin Therapy^a^

	Enrolled Patients (n = 95)				
	Baseline	12 mo	Change	Percent Change	*P* Value
Laboratory result					
Cholesterol, mg/dL					
Total^b^	187.4 (44.6)	144.0 (28.5)	-43.4	-23.2	<0.001
LDL^b^	119.9 (37.0)	80.1 (23.0)	-39.8	-33.2	<0.001
HDL^c^	47.7 (13.5)	48.0 (10.2)	0.3	0.6	0.293
TG, mg/dL^c^	139.7 (74.2)	121.4 (57.3)	-18.3	-13.1	0.009
Physiological result					
Pd/Pa^b^	0.87 (0.06)	0.88 (0.07)	0.01	1.2	0.242
FFR^b^	0.88 (0.05)	0.87 (0.06)	-0.01	-1.1	0.694
CFR^c^	4.06 (2.14)	4.17 (2.25)	0.11	2.7	0.626
IMR^c^	17.49 (9.35)	19.44 (12.11)	1.95	11.1	0.779
Imaging result					
Vessel, mm^2,^^c^	13.41 (4.55)	12.72 (4.31)	-0.69	-5.1	<0.001
Lumen, mm^2,^^c^	3.87 (1.28)	3.72 (1.20)	-0.15	-3.9	0.029
Atheroma, mm^2,^^c^	9.52 (4.00)	9.00 (3.79)	-0.52	-5.5	<0.001
PAV, %^c^	55.92 (7.30)	54.86 (7.63)	-1.06	-1.9	0.006
TAV_normalized_, mm^3,^^c^	145.0 (58.0)	135.2 (49.9)	-9.8	-6.8	<0.001
Remodeling index^c^	0.93 (0.17)	0.90 (0.17)	-0.03	-3.2	0.010
VH-IVUS					
Fibrous tissue, mm^2,^^c^	3.91 (2.07)	3.57 (2.01)	-0.34	-8.6	0.001
Fibrofatty, mm^2,^^c^	1.09 (0.87)	1.03 (0.94)	-0.06	-5.5	0.089
Necrotic core, mm^2,^^c^	1.19 (0.92)	1.15 (0.86)	-0.04	-3.3	0.856
Calcium, mm^2,^^b^	0.46 (0.52)	0.45 (0.55)	-0.01	-2.1	0.781

Values are n (%).

CFR = coronary flow reserve; FFR = fractional flow reserve; HDL = high-density lipoprotein; IMR = index of microcirculatory resistance; LDL = low-density lipoprotein; Pa = proximal aortic pressure; PAV = percent atheroma volume; Pd = distal arterial pressure; TAV = total atheroma volume; TG = triglycerides. VH-IVUS = virtual histology intravascular ultrasound.

a Intravascular ultrasound images were assessed at the minimal lumen site.

b Differences between baseline and 12-mo follow-up data were compared using the paired *t* test.

c Differences between baseline and 12-mo follow-up data were compared using the Wilcoxon signed rank-sum test.

## Results

### Characteristics of the study patients, lesions, and laboratory results

Between September 2013 and January 2018, a total of 119 patients were included in this study. Of these, 95 patients who completed coronary angiography and the physiological and imaging evaluations during the index procedure and after 12 months were enrolled in the final analysis. The baseline and lesion characteristics of the study population are shown in Table 1. At 12 months, mean atorvastatin dose was 42.7 mg and the percent change in LDL-C was -33.2% (baseline vs 12 months, 119.9 ± 37.0 vs 80.1 ± 23.0; *P* < 0.001) (Table 2). Thirty-three patients (34.7%) had an LDL-C <70 mg/dL, and 19 patients (20.0%) had a ≥50% LDL-C level reduction from baseline. Supplemental Figure 1 shows the distribution of the coronary angiographic, physiological, and IVUS measurements.

### Changes in coronary physiology and plaque parameters

**Figure 2:**
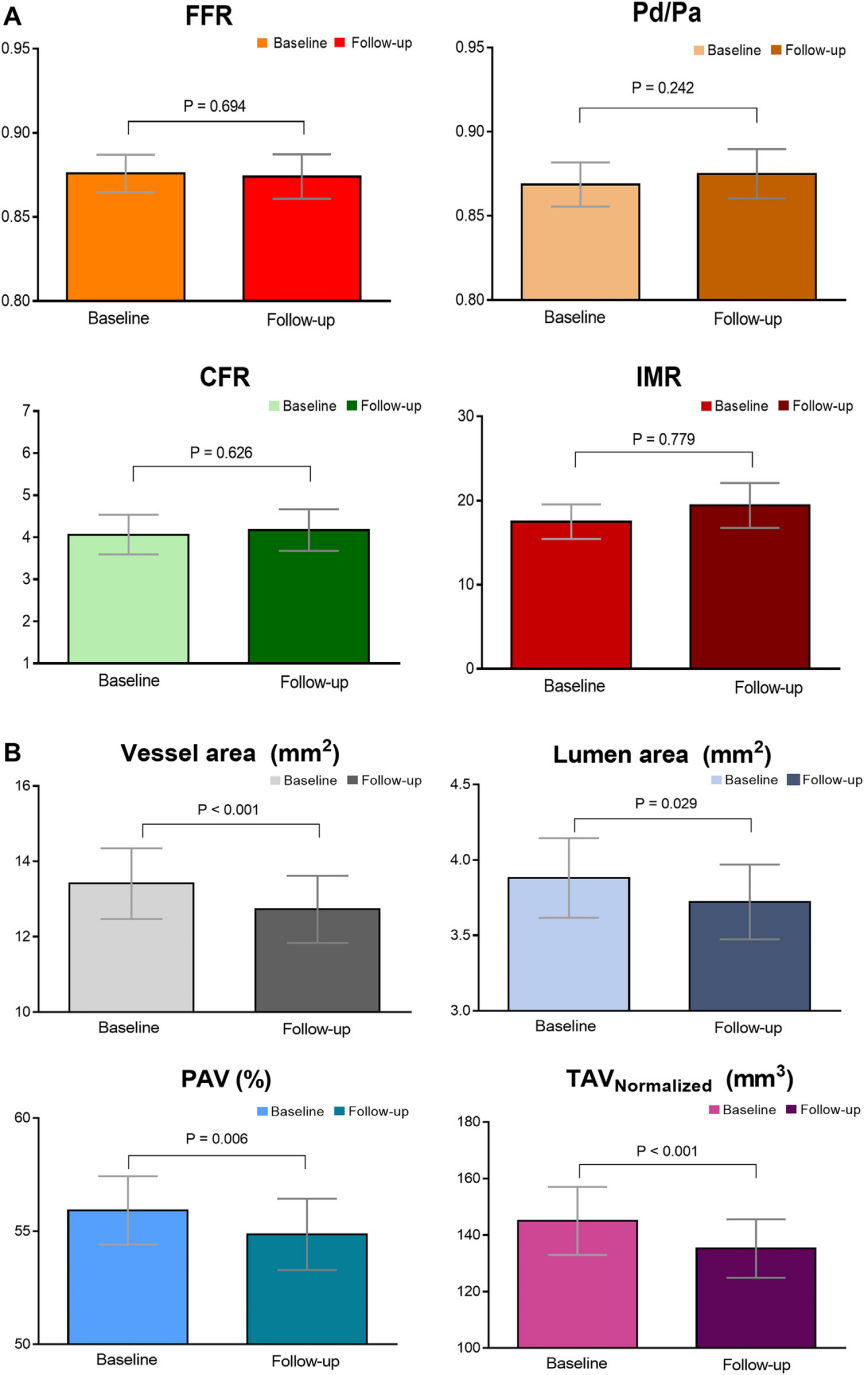
Changes in Coronary Flow and Plaque Parameters After Atorvastatin Therapy The changes in physiological **(A)** and intravascular imaging **(B)** parameters during the 12-mo atorvastatin therapy are shown. CFR = coronary flow reserve; IMR = index of microcirculatory resistance; Pa = proximal aortic pressure; PAV = percent atheroma volume; Pd = distal coronary pressure; TAV = total atheroma volume; other abbreviations as in Figure 1.

There was no difference in the mean FFR values with 12-month atorvastatin therapy (0.88 ± 0.05 vs 0.87 ± 0.06; *P* = 0.694), and other physiological parameters including resting Pd/Pa, IMR, and CFR also did not show any significant differences. IVUS parameters measured at the MLA site, the mean values of the vessel area (13.41 ± 4.55 mm^2^ vs 12.72 ± 4.31 mm^2^; *P* < 0.001), lumen area (3.87 ± 1.28 mm^2^ vs 3.72 ± 1.20 mm^2^; *P* = 0.029), and atheroma area (9.52 ± 4.00 mm^2^ vs 9.00 ± 3.79 mm^2^; *P* < 0.001) decreased after 12 months. In the volumetric analysis of the IVUS findings, both PAV (55.92 ± 7.30% vs 54.86 ± 7.63%; *P* = 0.006) and normalized TAV (145.0 ± 58.0 mm^3^ vs 135.2 ± 49.9 mm^3^; *P* < 0.001) were significantly lower after 12-month atorvastatin therapy (Table 2, Figure 2).

### Correlation and determinants of FFR changes

**Figure 3:**
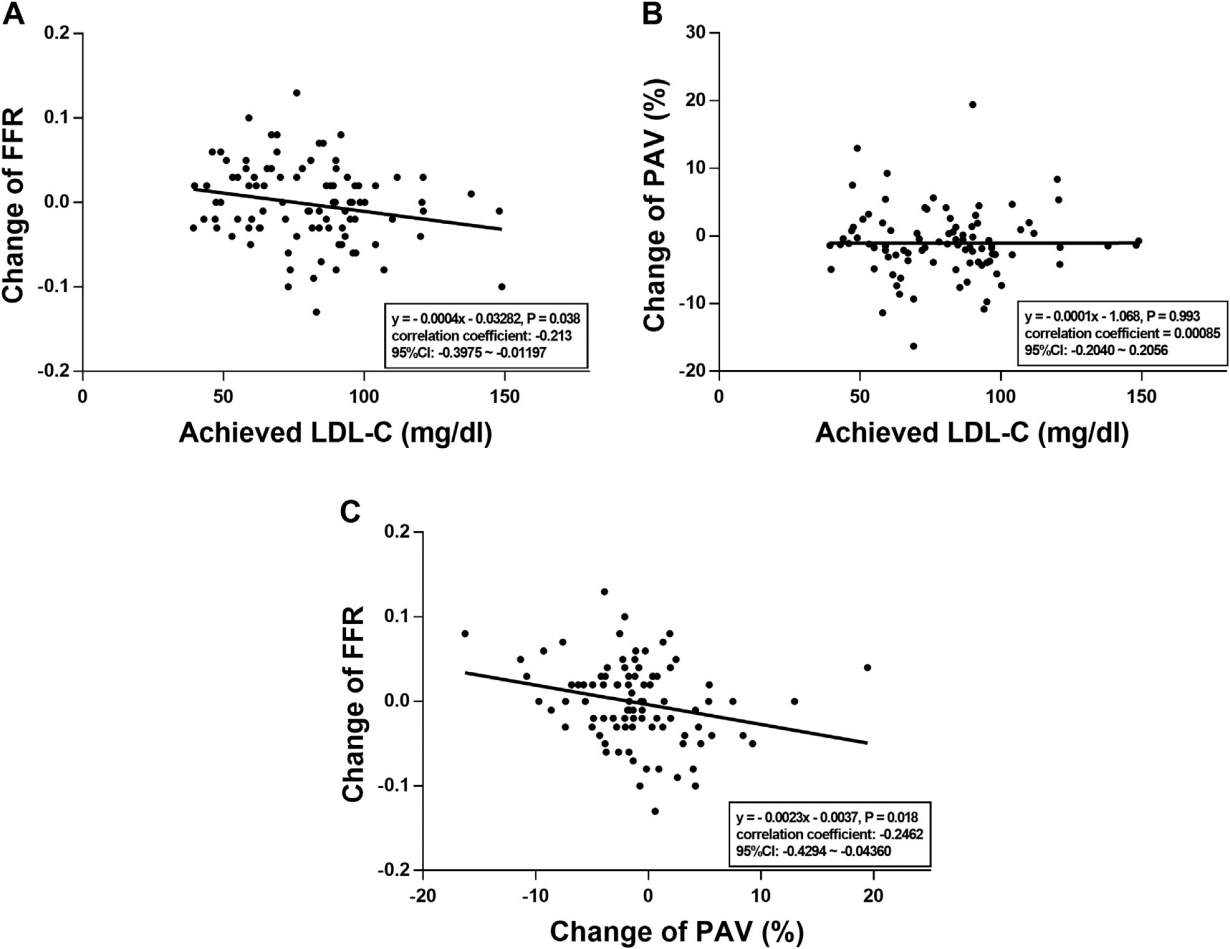
Correlations between Coronary Flow, Plaque, and Achieved LDL-C Level The correlations between change in FFR and achieved LDL-C level **(A)**, change in PAV and achieved LDL-cholesterol level **(B)**, and change in FFR and change in PAV **(C)** are shown for the 12-mo atorvastatin therapy. LDL-C = low-density lipoprotein cholesterol; other abbreviations as in Figures 1 and 2.

There was an inverse correlation between the change in the FFR and LDL-C level achieved (correlation coefficient: -0.213; 95% CI: -0.001-0.067; *P* = 0.038) and between the change in the FFR and PAV (correlation coefficient: -0.246; 95% CI: -0.014-0.006; *P* = 0.018) (Figure 3). In a multivariable logistic regression analysis, in model 1 analyzed with clinical and anatomical parameters, acute coronary syndrome (odds ratio [OR]: 2.54; 95% CI: 1.01-6.39; *P* = 0.047), male gender (OR: 0.28; 95% CI: 0.09-0.84; *P* = 0.024), and use of angiotensin-converting enzyme inhibitor or angiotensin II receptor blockers (OR: 0.27; 95% CI: 0.09-0.76; *P* = 0.013) are independent correlates of decreased FFR during 12 months. In model 2 analyzed with parameters of model 1 and change of laboratory, physiological, and imaging parameters, the change in PAV was the only independent predictor of a decrease in FFR (OR: 1.32; 95% CI: 1.01-1.73; *P* = 0.041) (Supplemental Table 2).

### Changes in coronary physiology and plaque parameters according to the change in LDL-C

**Figure 4:**
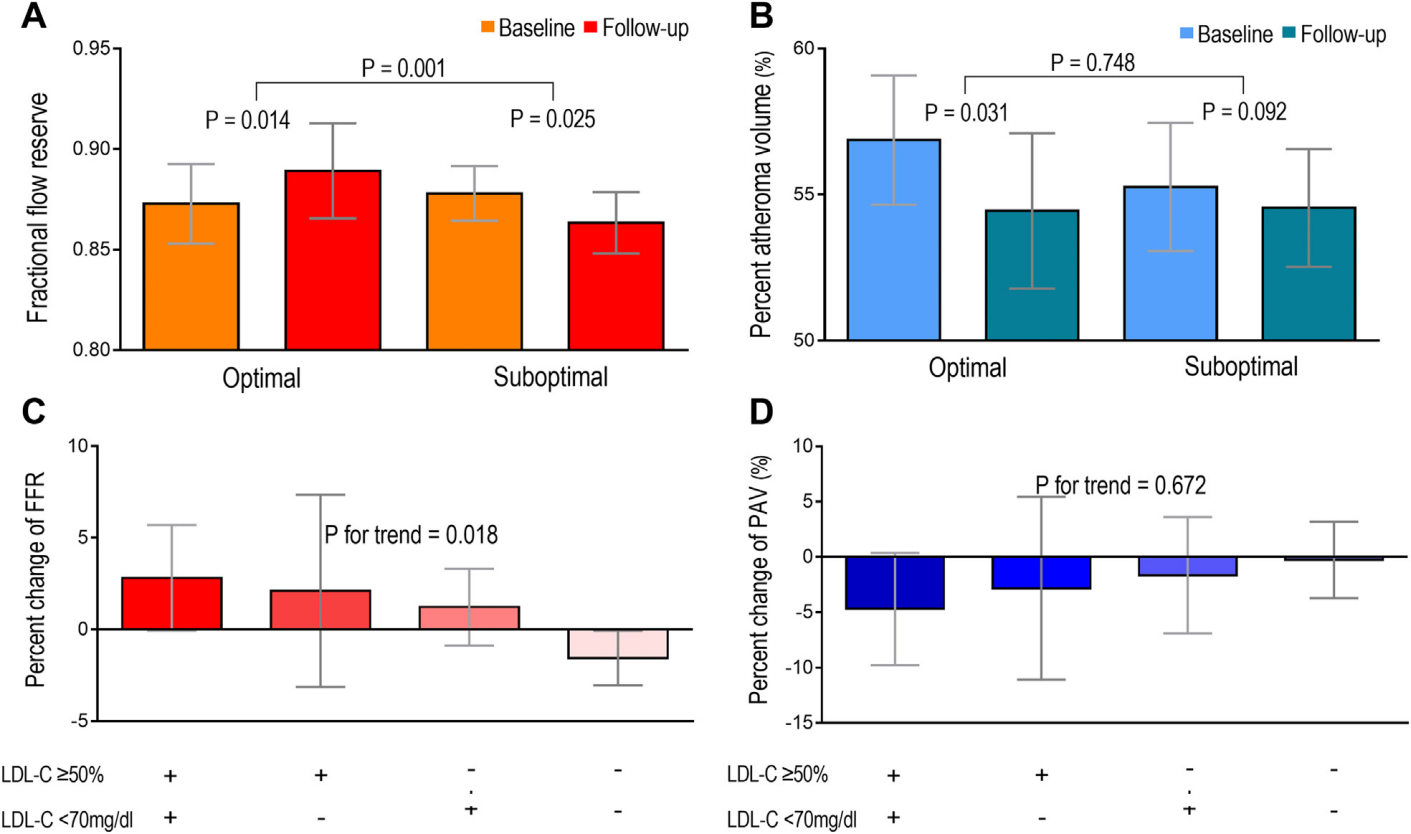
Changes in Coronary Flow and Plaque by Response to Atorvastatin Therapy In each figure, the changes in the FLR and PAV are shown according to the response in the 12-mo atorvastatin therapy. The change in FFR **(A)** and PAV **(B)** according to the response to the atorvastatin therapy. The percent change in the FFR **(C)** and PAV **(D)** according to the response to the atorvastatin therapy. ∗Optimal treatment goal: LDL-C <70 mg/dL or ≥50% LDL-C reduction compared with baseline. Abbreviations as in Figures 1, 2, and 3.

According to the achieved LDL-C level to atorvastatin therapy, the FFR value was significantly increased (0.87 ± 0.06 vs 0.89 ± 0.07; *P* = 0.014) in the optimal treatment group, the vessel area and atheroma area were decreased, and the lumen area was preserved. Furthermore, the PAV (56.81% ± 6.44% vs 55.18% ± 8.19%; *P* = 0.031) and normalized TAV (152.6 ± 65.5 mm^3^ vs 139.4 ± 53.9 mm^3^; *P* = 0.032) were significantly decreased. In the suboptimal treatment group, the FFR (0.88 ± 0.05 vs 0.86 ± 0.06; *P* = 0.025) and the lumen area (3.90 ± 1.34 mm^2^ vs 3.73 ± 1.31 mm^2^; *P* = 0.017) were significantly decreased. The PAV (55.28% ± 7.85% vs 54.64% ± 7.29%; *P* = 0.092) did not significantly change in this group. The change of FFR according to the response to atorvastatin therapy showed a statistically significant difference between baseline and 12-month follow-up (*P* = 0.001) (Table 3, Figures 4A and 4B). When comparing the responses depending on the achievement of ≥50% LDL-C reduction from baseline or LDL-C <70 mg/dL, statistically significant incremental response in the percent change of FFR was demonstrated, but just a numerical trend in the percent change of PAV (Figures 4C and 4D). Analysis of coronary physiological and plaque parameters according to atorvastatin intensity is shown in Supplemental Table 3. CFR was significantly increased after high-intensity atorvastatin therapy (3.85 ± 2.37 vs 4.75 ± 2.69; *P* = 0.026), whereas CFR was decreased and IMR was increased after low-intensity atorvastatin therapy (4.22 ± 1.97 vs 3.74 ± 1.76; *P* = 0.041; 16.69 ± 7.70 vs 20.75 ± 12.06; *P* = 0.044, respectively) (Supplemental Figure 2). Vessel area, lumen area, atheroma volume, and remodeling index were significantly decreased after high-intensity statin therapy.

**Table 3:** Laboratory, Physiological, and Intravascular Imaging Results According to the Response to Atorvastatin Therapy^a^^,^^b^

	Optimal Treatment (n = 39)					Suboptimal Treatment (n = 56)				
	Baseline	12 mo	Change	Percent Change	*P* Value	Baseline	12 mo	Change	Percent Change	*P* Value
Laboratory result										
Cholesterol, mg/dL										
Total^c^	175.3 (50.8)	120.9 (17.9)	-54.4	-31.0	<0.001	195.3 (38.6)	159.2 (23.5)	-36.1	-18.5	<0.001
LDL^c^	114.8 (39.7)	59.8 (12.4)	-55.0	-47.9	<0.001	123.5 (34.9)	94.1 (17.5)	-29.4	-23.8	<0.001
HDL^d^	45.3 (12.5)	47.7 (10.1)	2.4	5.3	0.150	49.2 (14.1)	48.2 (10.4)	-1.0	-2.0	0.996
TG, mg/dL^d^	117.7 (42.6)	98.8 (36.2)	-18.9	-16.1	0.005	154.1 (86.4)	136.1 (63.7)	-18.0	-11.7	0.168
Physiological result										
Pd/Pa^c^	0.86 (0.07)	0.89 (0.08)	0.03	3.5	0.005	0.87 (0.06)	0.87 (0.06)	0.00	0.0	0.338
FFR^c^	0.87 (0.06)	0.89 (0.07)	0.02	2.3	0.014	0.88 (0.05)	0.86 (0.06)	-0.02	-2.3	0.025
CFR^d^	4.25 (1.97)	4.69 (2.61)	0.44	10.4	0.427	3.93 (2.26)	3.82 (1.91)	-0.11	-2.8	0.943
IMR^d^	16.33 (9.58)	15.91 (6.22)	-0.42	-2.6	0.809	18.27 (9.20)	21.82 (14.40)	3.55	19.4	0.743
Imaging result										
Vessel, mm^2,^^d^	13.88 (4.76)	13.07 (4.43)	-0.81	-5.8	0.001	13.08 (4.42)	12.49 (4.25)	-0.59	-4.5	0.003
Lumen, mm^2,^^d^	3.84 (1.20)	3.70 (1.02)	-0.14	-3.6	0.669	3.90 (1.34)	3.73 (1.31)	-0.17	-4.4	0.014
Atheroma, mm^2,^^d^	10.04 (4.13)	9.36 (3.90)	-0.68	-6.8	0.002	9.15 (3.90)	8.76 (3.73)	-0.39	-4.3	0.045
PAV, %^d^	56.81 (6.44)	55.18 (8.19)	-1.63	-2.9	0.031	55.28 (7.85)	54.64 (7.29)	-0.64	-1.2	0.092
TAV_normalized_, mm^3,^^d^	152.6 (65.5)	139.4 (53.9)	-13.2	-8.7	0.032	139.6 (52.2)	132.2 (47.2)	-7.4	-5.3	0.001
Remodeling index^d^	0.96 (0.14)	0.93 (0.15)	-0.03	-3.1	0.083	0.90 (0.18)	0.88 (0.18)	-0.02	-2.2	0.061
VH-IVUS										
Fibrous tissue, mm^2,^^d^	4.32 (2.36)	3.96 (2.22)	-0.36	-9.0	0.007	3.62 (1.81)	3.29 (1.83)	-0.33	-10.0	0.021
Fibrofatty, mm^2,^^d^	1.20 (0.93)	1.05 (0.89)	-0.15	-14.2	0.078	1.01 (0.82)	1.01 (0.99)	0.00	0.0	0.417
Necrotic core, mm^2,^^d^	1.14 (0.95)	1.14 (0.88)	0.00	0.0	0.940	1.23 (0.90)	1.17 (0.85)	-0.06	-5.1	0.750
Calcium, mm^2,^^c^	0.35 (0.37)	0.37 (0.45)	0.02	5.4	0.647	0.54 (0.59)	0.51 (0.61)	-0.03	-5.8	0.457

Values are n (%).

Abbreviations as in Table 2.

a Intravascular ultrasound images were assessed at the minimal lumen site.

b Optimal treatment goal: LDL-C <70 mg/dL or ≥50% LDL-C reduction compared with baseline.

c Differences between baseline and 12-mo follow-up data were compared using the paired *t* test.

d Differences between baseline and 12-mo follow-up data were compared using the Wilcoxon signed rank-sum test.

### Clinical outcomes and adverse events with atorvastatin therapy

At 1 year, the rate of major cardiac adverse events was 4 of 95 patients (4.2%). All events were repeat revascularizations and, of those, 2 were target vessel revascularizations. Adverse event and drug compliance data are shown in Supplemental Tables 4 and 5.

## Discussion

The major findings of the present study, which investigated the effects of atorvastatin therapy on the serial changes in coronary flow and plaque parameters in patients with intermediate CAD, were that: 1) after the 12-month atorvastatin therapy, the FFR and other coronary physiological parameters were not significantly changed whereas the atheroma volume represented by PAV and normalized TAV was decreased with negative remodeling of the target vessel; 2) the changes in PAV and the achieved LDL-C levels showed significant inverse correlations with FFR changes; and 3) in patients who achieved the optimal LDL-C target, the FFR increased and the PAV decreased, whereas in patients who did not, the FFR decreased and the PAV was unchanged. According to the results of the current study that an optimal LDL-C reduction with atorvastatin therapy causes beneficial changes in coronary physiology and plaques, it is necessary to thoroughly follow the current guidelines as directed for LLT for cholesterol treatment.

For favorable outcomes in patients with deferred angioplasty based on FFR, optimal medical therapy is highly recommended,^2^^,^^7^^,^^14^, ^15^, ^16^, ^17^, ^18^, ^19^ and previous studies have reported that statin-based intensive LLT leads to plaque regression or stabilization.^3^^,^^10^^,^^11^ Although the effects of high-intensity statin therapies on clinical outcomes and morphologic plaque changes are well known, their effects on coronary physiological changes have not been sufficiently validated. In the YELLOW (Reduction in Yellow Plaque by Intensive Lipid Lowering Therapy) trial,^9^ which observed FFR short-term changes, the group with intensive rosuvastatin therapy showed only a trend of FFR increase without statistical significance (intensive vs standard, 0.75 ± 0.1 vs 0.73 ± 0.1; *P* = 0.360), and could not modify the plaque volume (normalized TAV mm^3^: baseline intensive, 195.8 ± 63.3; follow-up intensive, 209.6 ± 74.1), which might be related to a short-term follow-up (6 to 8 weeks). A recent study using computed tomography–derived FFR reported that rosuvastatin therapy leads to physiological gain in patients with intermediate CAD.^20^ However, this study used only noninvasive measurements, and follow-up LDL-C levels were unchanged even after rosuvastatin therapy (baseline vs follow-up, 3.52 [IQR: 2.92-4.67] vs 3.89 [IQR: 3.10-4.12] mmol/L; *P* = 0.45); it is difficult to explain by which mechanism this optimal LLT caused physiological changes. The current study exactly shows the real effects of atorvastatin therapy and LDL-C modification on coronary anatomy and physiology using invasive coronary anatomical and physiological measurements.

**Figure 5:**
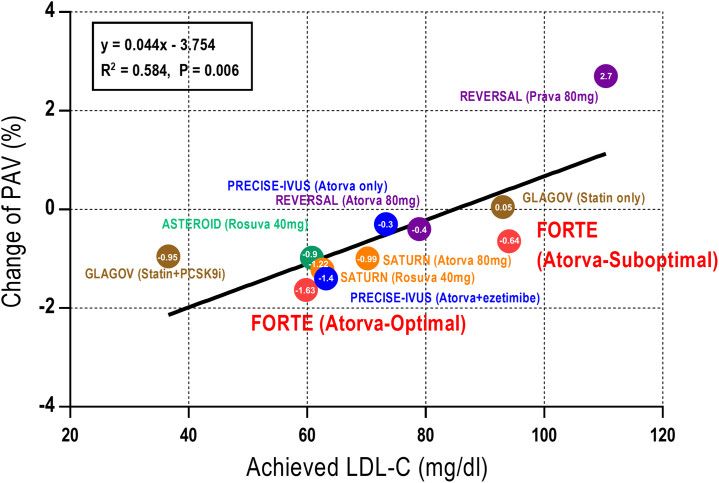
Relationship Between Achieved LDL-C Levels and Change in PAV The change of PAV was significantly dependent on achieving the LDL-C goal, shown as the results with previous historical intravascular ultrasound studies. ASTEROID = A Study to Evaluate the Effect of Rosuvastatin on Intravascular Ultrasound–Derived Coronary Atheroma Burden; Atorva = atorvastatin; GLAGOV = Global Assessment of Plaque Regression With a PCSK9 Antibody as Measured by Intravascular Ultrasound; PRECISE-IVUS = Plaque Regression With Cholesterol Absorption Inhibitor or Synthesis Inhibitor Evaluated by Intravascular Ultrasound; Prava = pravastatin; REVERSAL = Reversal of Atherosclerosis With Aggressive Lipid-Lowering; Rosuva = Rosuvastatin; SATURN = Study of Coronary Atheroma by Intravascular Ultrasound: Effect of Rosuvastatin versus Atorvastatin; other abbreviations as in Figures 2 and 3.

In previous studies using serial intravascular imaging to demonstrate plaque changes, high-intensity atorvastatin or rosuvastatin therapy negatively remodeled the vessel mainly by decreasing the TAV and PAV while increasing or decreasing the lumen size in some cases.^3^^,^^10^^,^^11^^,^^21^ Similarly, the TAV and PAV significantly decreased in this study, and negative remodeling of the vessel including the lumen area was observed after the 12-month atorvastatin therapy. The change of PAV was significantly dependent on achieving the LDL-C goal, shown as the results with previous IVUS studies (Figure 5). The mean FFR value and other physiological parameters did not change significantly in overall patients, indicating a preserved coronary flow during 12 months. Furthermore, when the changes in coronary flow and plaque were compared according to the achievement of the LDL-C target, the results differed. In the group that had achieved the optimal LDL-C target using atorvastatin therapy, the lumen area was well preserved due to a significant decrease in plaque volume despite negative remodeling of the vessel and increased FFR with improving trend of CFR. By contrast, in patients who did not achieve the LDL-C target, the coronary flow eventually decreased because of the narrowed lumen related to insufficient reduction in the coronary plaque and impaired trend of microvascular function (Central Illustration). These distinct changes in coronary anatomy and physiology may support the results of previous studies that an optimal LLT improves the long-term clinical outcomes^8^^,^^15^ and the favorable clinical outcome in the medical therapy group of the ISCHEMIA (International Study of Comparative Health Effectiveness With Medical and Invasive Approaches) trial.^22^ In the responses depending on the achievement of ≥50% LDL-C reduction from baseline or LDL-C <70 mg/dL, statistically significant incremental response in the percent change of FFR and numerical trend in the percent change of PAV were observed, especially with dominant importance of LDL-C ≥50% reduction (Figure 4). These responses can strongly support the current updated guidelines for cholesterol treatment.

**Central Illustration:**
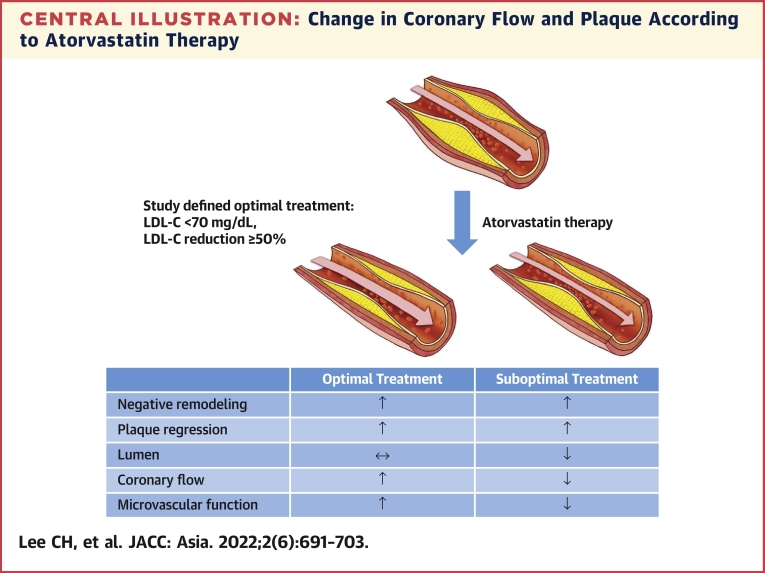
Change in Coronary Flow and Plaque According to Atorvastatin Therapy In the patients who achieved the optimal LDL-C target by atorvastatin therapy, the lumen area was well preserved due to a significant decrease in plaque volume despite negative remodeling of the vessel and increased FFR with improving trend of microvascular function, whereas this was not the case in patients who failed to reach the target level. ↑, increased; ↔, unchanged; ↓, decreased. FFR = fractional flow reserve; LDL-C = low-density lipoprotein cholesterol.

There are several debatable points in this study. The impact of LLT on reducing cardiovascular events cannot be fully explained by coronary physiological and plaque burden modification alone. Because the current study was performed with IVUS, which has lower resolution power than optical coherence tomography, there were limitations to show the results related to the vulnerability or cap thickness of plaque as in the previous studies.^23^^,^^24^ In the current VH-IVUS analysis, the changes in thin-cap fibroatheroma and necrotic core showed a numerically decreased trend similar to the previous VH-IVUS study,^25^ without statistical significance, which might be related to a relatively mild to moderate plaque and a small number of cases.

The change in coronary physiological parameters according to the plaque composition could not be revealed. These results were similar to the FIRST (Fractional Flow Reserve and Intravascular Ultrasound Relationship Study),^26^ demonstrating that MLA correlated with FFR and plaque characteristics had no correlation with FFR. Another interesting issue is the change of microvascular parameters such as CFR and IMR according to statin intensity. Although there are limitations to interpretation with small numbers, parameters reflecting the epicardial coronary environment, such as FFR and PAV, were related to the achieved LDL-C target goal, and parameters reflecting the microcirculatory environment, such as CFR and IMR, were related to the intensity of atorvastatin (Supplemental Figure 2), and similar trends were observed in another study.^27^

Another interesting finding was that the physiological coronary vascular response was bidirectional, ie, the FFR was decreased or increased, according to whether the LDL-C target had been achieved after the atorvastatin therapy. However, the anatomic coronary vascular response based on IVUS parameters was unidirectional, ie, the target achievement was reflected in the degree of decrease. Therefore, as a surrogate marker for assessing the vascular response to a certain therapy, the coronary physiological parameter can be useful in addition to traditional anatomical parameters. Furthermore, changes in FFR had a significant inverse correlation with changes in PAV and achieved LDL-C levels (Figure 3), confirming that coronary anatomical and physiological responses were highly correlated after atorvastatin therapy.

Although the updated lipid guidelines recommend a more powerful LDL-C modification therapy, especially in very high-risk patients,^5^^,^^6^ the number of patients that fail to reach the optimal LDL-C target is not small in real-world practice.^28^, ^29^, ^30^ Considering the results of the current study showing the beneficial changes in FFR and coronary plaques by LDL-C modification, a more aggressive LLT should be emphasized, and the current cholesterol treatment guidelines should be thoroughly followed.

### Study limitations

Several limitations should be considered. First, the current study was not free from selection bias; it was not a randomized trial and included a limited number of patients. Second, about half of all patients’ conditions were due to various causes unable to achieve a titration with high-intensity doses. Therefore, fewer patients may have been treated optimally. However, this can be seen as a result reflecting daily practice. Third, the current study did not follow the latest updated guidelines because of the timing of the study. Forth, the coronary physiological gain and correlation between PAV or LDL-C was small even with optimal lipid-lowering treatment during 12 months and the clinical impact may be questioned. Fifth, because the independent predictor of decreased FFR is not a result derived from multiple linear regression due to the absolute value or change of FFR being small, there may be limitations in interpretation of the results. However, the long-term impact may be greater because atherosclerosis has the characteristic of accumulating. Despite these limitations, our data clearly demonstrated the coronary anatomical and physiological changes after atorvastatin therapy and these findings may facilitate the generation of hypotheses for future research.

## Conclusions

In patients with intermediate CAD who received atorvastatin therapy for 12 months, the coronary plaque showed significantly decreased atheroma volume whereas the coronary flow was unchanged according to the FFR. Patients who achieved the LDL-C target had significantly increased FFR and decreased PAV values, whereas this was not the case in patients who failed to reach the target level.

Perspectives **COMPETENCY IN MEDICAL KNOWLEDGE:** In patients with intermediate CAD, atorvastatin therapy showed a decrease in plaque with negative remodeling of the vessel, and the coronary flow did not change. However, in patients achieving an optimal LDL-C, not only was there a decrease in plaque but also an increase in the coronary flow was observed. High-intensity atorvastatin therapy improved microvascular function.

**TRANSLATIONAL OUTLOOK:** Future large-scale randomized research would be needed to confirm the target goal of a LLT for the proper changes in the coronary flow and plaque in patients with CAD.

## Funding Support and Author Disclosures

Dr Nam has received institutional research grant support from Pfizer (Viatris). Drs Koo and Lee have received institutional research grant support from St. Jude Medical (Abbott Vascular) and Philips Volcano. Dr Doh has received institutional research grant support from Philips Volcano. The companies had no role in the study design, conduct, data analysis, or manuscript preparation. All other authors have reported that they have no relationships relevant to the contents of this paper to disclose.
