# Supplementary material for: Effect of Atorvastatin on Serial Changes in Coronary Physiology and Plaque Parameters
Source: JACC Asia. 2022 Nov 1;2(6):691–703. doi: 10.1016/j.jacasi.2022.07.010 (PMC9700034; doi:10.1016/j.jacasi.2022.07.010)

**Supplemental Data**

Lee CH, et al. “Effect of Atorvastatin on Serial Changes in Coronary Physiology and Plaques Parameters”

**Supplemental Table 1.** Inclusion and exclusion Criteria

**Supplemental Table 2.** Independent Predictor of Decreased Fractional Flow Reserve Change during Atorvastatin Therapy

**Supplemental Table 3.** Laboratory, Physiologic, and Intravascular Imaging Results According to the Dose of Atorvastatin Therapy

**Supplemental Table 4.** Adverse event

**Online Table 5.** Drug Adherence

**Supplemental Figure 1**. Histograms of Coronary Angiographic, Physiologic, and Intravascular Imaging Measurements

Abbreviations: CFR, coronary flow reserve; DS, diameter stenosis; FFR, fractional flow reserve; IMR, index of microcirculatory resistance; MLA, minimal lumen area; PAV, percent atheroma volume.

**Supplemental Figure 2.** Changes in Coronary Flow Reserve and Index of Microcirculatory Resistance According to the Dose of Atorvastatin Therapy

**Supplemental Table 1.** Inclusion and exclusion Criteria

| Inclusion criteria |
| --- |
| - Patient > 18 years of age and willing to participate - Patients with intermediate CAD (30-80% diameter stenosis by visual estimation) on angiography with a FFR>0.80, or who had non-culprit CAD for which revascularization was not planned - Signed written Informed Consent |
| Exclusion criteria |
| - Patients who are in cardiogenic shock - Patients with LVEF<35% - Patients with left main disease, restenotic, bypass grafted lesions - Patients with platelet count < 100,000 cell/mm^3^ - Patients with co-morbidity that reduces life expectancy to one year - Patients with a history of stroke or transient ischemic attack within 6 months - Patients for whom discontinuation of medication is planned due to surgery - Patients with known adverse reaction to HMG CO-A reductase therapy (statins) - Patients with liver disease (elevation of AST or ALT more than 2 times) - Patient with creatinine > 2.0 mg/dL - Pregnant women and women of childbearing potential who intend to have children during the duration of the trial - Patients who consistently must take medications affecting lipid levels in blood except the investigational product |

**Supplemental Table 2.** Independent Predictor of Decreased Fractional Flow Reserve Change during Atorvastatin Therapy

| **Variables** | **OR (95% CI)** | **Wald Chi-Square** | **P Value** |
| --- | --- | --- | --- |
| Model 1 |  |  |  |
| Acute coronary syndrome | 2.54 (1.01–6.39) | 3.95 | 0.047 |
| Male gender | 0.28 (0.09–0.84) | 5.11 | 0.024 |
| ACE inhibitor or ARB | 0.27 (0.09–0.76) | 6.13 | 0.013 |
| Model 2 |  |  |  |
| Change of PAV | 1.32 (1.01-1.73) | 4.16 | 0.041 |

Abbreviations: CI, confidence interval; OR, odds ratio; other abbreviations are as in Table 1 and 2.

**Supplemental Table 3.** **Laboratory, Physiologic, and Intravascular Imaging Results According to the Dose of Atorvastatin Therapy**

| **Characteristic** | **High intensity (n=47)** | | | | | |  | **Low intensity (n=48)** | | | | |
| --- | --- | --- | --- | --- | --- | --- | --- | --- | --- | --- | --- | --- |
|  | **Baseline** | **12 months** | **Change** | **Percent change** | ***P*** | **Baseline** | | | **12 months** | **Change** | **Percent change** | ***P*** |
| **Laboratory result** |  |  |  |  |  |  | | |  |  |  |  |
| Cholesterol (mg/dl) |  |  |  |  |  |  | | |  |  |  |  |
| Total^a^ | 194.9 (44.2) | 147.5 (28.9) | -47.4 | -24.3% | <0.001 | 180.0 (44.3) | | | 140.6 (27.9) | -39.4 | -21.8% | <0.001 |
| LDL^a^ | 126.8 (37.1) | 83.4 (22.4) | -43.4 | -34.2% | <0.001 | 113.2 (36.0) | | | 76.8 (23.3) | -36.4 | -32.1% | <0.001 |
| HDL^b^ | 47.0 (11.7) | 47.2 (11.7) | 0.2 | 0.4% | 0.863 | 48.3 (15.3) | | | 48.7 (8.7) | 0.4 | 0.8% | 0.862 |
| TG (mg/dl)^b^ | 142.2 (72.2) | 123.5 (59.8) | -18.7 | -13.1% | 0.065 | 137.3 (76.7) | | | 119.3 (55.3) | -18.0 | -13.1% | 0.129 |
| **Physiologic result** |  |  |  |  |  |  | | |  |  |  |  |
| Pd/Pa^a^ | 0.86 (0.05) | 0.87 (0.07) | 0.01 | 1.1% | 0.211 | 0.87 (0.06) | | | 0.87 (0.06) | 0.00 | 0.0% | 0.606 |
| FFR^a^ | 0.87 (0.05) | 0.87 (0.07) | 0.00 | 0.0% | 0.975 | 0.88 (0.05) | | | 0.87 (0.06) | -0.01 | -1.1% | 0.612 |
| CFR^b^ | 3.85 (2.37) | 4.75 (2.69) | 0.90 | 23.3% | 0.026 | 4.22 (1.97) | | | 3.74 (1.76) | -0.48 | -11.3% | 0.041 |
| IMR^b^ | 18.55 (11.22) | 17.69 (12.13) | -0.86 | -4.6% | 0.593 | 16.69 (7.70) | | | 20.75 (12.06) | 4.06 | 24.3% | 0.044 |
| **Imaging result** |  |  |  |  |  |  | | |  |  |  |  |
| Vessel (mm^2^)^b^ | 13.62 (4.29) | 12.61 (4.27) | -1.01 | -7.4% | <0.001 | 13.20 (4.83) | | | 12.83 (4.39) | -0.37 | -2.8% | 0.048 |
| Lumen (mm^2^)^b^ | 3.88 (1.31) | 3.71 (1.25) | -0.17 | -4.3% | 0.034 | 3.87 (1.26) | | | 3.73 (1.16) | -0.14 | -3.6% | 0.262 |
| Atheroma (mm^2^)^b^ | 9.73 (3.84) | 8.90 (3.76) | -0.83 | -8.5% | <0.001 | 9.31 (4.17) | | | 9.10 (3.86) | -0.21 | -2.2% | 0.164 |
| PAV (%)^b^ | 56.05 (8.24) | 54.73 (7.82) | -1.32 | -2.3% | 0.079 | 55.78 (6.31) | | | 55.00 (7.52) | -0.78 | -1.3% | 0.301 |
| TAV_normalized_ (mm^3^)^b^ | 140.9 (57.2) | 135.4 (49.7) | -5.5 | -3.9% | 0.283 | 149.0 (59.3) | | | 135.0 (50.6) | -14.0 | -9.3% | 0.025 |
| Remodeling index^b^ | 0.92 (0.16) | 0.86 (0.15) | -0.06 | 6.5% | <0.001 | 0.94 (0.18) | | | 0.93 (0.18) | -0.01 | -1.0% | 0.680 |
| **VH-IVUS** |  |  |  |  |  |  | | |  |  |  |  |
| Fibrous tissue (mm^2^)^b^ | 3.83 (1.68) | 3.29 (1.73) | -0.54 | -14.0% | 0.001 | 3.98 (2.40) | | | 3.83 (2.24) | -0.15 | -3.7% | 0.374 |
| Fibrofatty (mm^2^)^b^ | 1.20 (0.86) | 0.97 (0.90) | -0.23 | -19.1% | 0.012 | 0.98 (0.86) | | | 1.08 (0.99) | 0.10 | 10.2% | 0.349 |
| Necrotic core (mm^2^)^b^ | 1.21 (0.87) | 1.26 (0.94) | 0.05 | 4.1% | 0.612 | 1.18 (0.97) | | | 1.05 (0.77) | -0.13 | -11.0% | 0.175 |
| Calcium (mm^2^)^a^ | 0.54 (0.61) | 0.55 (0.64) | 0.01 | 1.8% | 0.772 | 0.39 (0.40) | | | 0.35 (0.44) | -0.04 | -10.2% | 0.484 |

Data are shown as the mean (SD) for continuous variables and absolute number (percentage) for dichotomous variables.

*High intensity: Atorvastatin 40 mg, 80mg; Low intensity: Atorvastatin 20 mg

^a^Differences between baseline and 12-month follow-up data were compared using the paired t-test.

^b^Differences between baseline and 12-month follow-up data were compared using the Wilcoxon signed rank-sum test.

Abbreviations: CFR, coronary flow reserve; FFR, fractional flow reserve; HDL, high-density lipoprotein; IMR, index of microcirculatory resistance; LDL, low-density lipoprotein; Pa, proximal aortic pressure; Pd, distal arterial pressure; TG, triglycerides.

**Supplemental Table 4. Adverse Events**

| **Characteristics** | | **All patients**  **(n = 95)** | **High intensity**  **(n = 47)** | **Low intensity**  **(n = 48)** | **P Value** |
| --- | --- | --- | --- | --- | --- |
| Any AE | 38 (40.0) | | 17 (36.2) | 21 (43.8) | 0.586 |
| Cardiac | 4 (4.2) | | 1 (2.1) | 3 (6.2) | 0.625 |
| Non-Cardiac | 34 (35.8) | | 14 (29.8) | 20 (41.7) | 0.320 |
| Myalgia | 5 (5.3) | | 2 (4.3) | 3 (6.2) | 0.998 |
| LFT elevation | 3 (3.2) | | 0 (0.0) | 3 (6.2) | 0.248 |
| GI trouble | 5 (5.3) | | 2 (4.3) | 3 (6.2) | 0.999 |
| Malignancy | 1 (1.1) | | 1 (2.1) | 0 (0.0) | 0.992 |
| Drug discontinuation | 1 (1.1) | | 0 (0.0) | 1 (2.1) | 0.997 |

* Data are shown as mean (SD) for continuous variables and absolute numbers (percentage) for dichotomous variables.

**LFT elevation: liver function test > 3 times of upper normal limit

Abbreviations: AE, adverse event; LFT, liver function test; GI, gastrointestinal

**Supplemental Table 5. Drug adherence**

|  | **All patients**  **(n=95)** | **High intensity (n=47)** | **Low intensity (n=48)** | **P value** |
| --- | --- | --- | --- | --- |
| PDC | 89.2±30.8 | 88.5±35.8 | 89.9±25.2 | 0.829 |
| CMG | 6.4±18.5 | 7.4±21.1 | 5.5±15.6 | 0.626 |
| CMA | 84.5±29.5 | 82.6±35.9 | 86.4±21.6 | 0.544 |
| MRA | 98.4±4.0 | 97.8±5.1 | 99.0±2.4 | 0.138 |

Abbreviations: PDC, Proportion of days covered; CMG, Continuous measure of medication gap; CMA, Continuous measure of medication acquisition; MRA, Medication refill adherence

**Supplemental Figure 1.** **Histograms of Coronary Angiographic, Physiologic, and Intravascular Imaging Measurements**

**
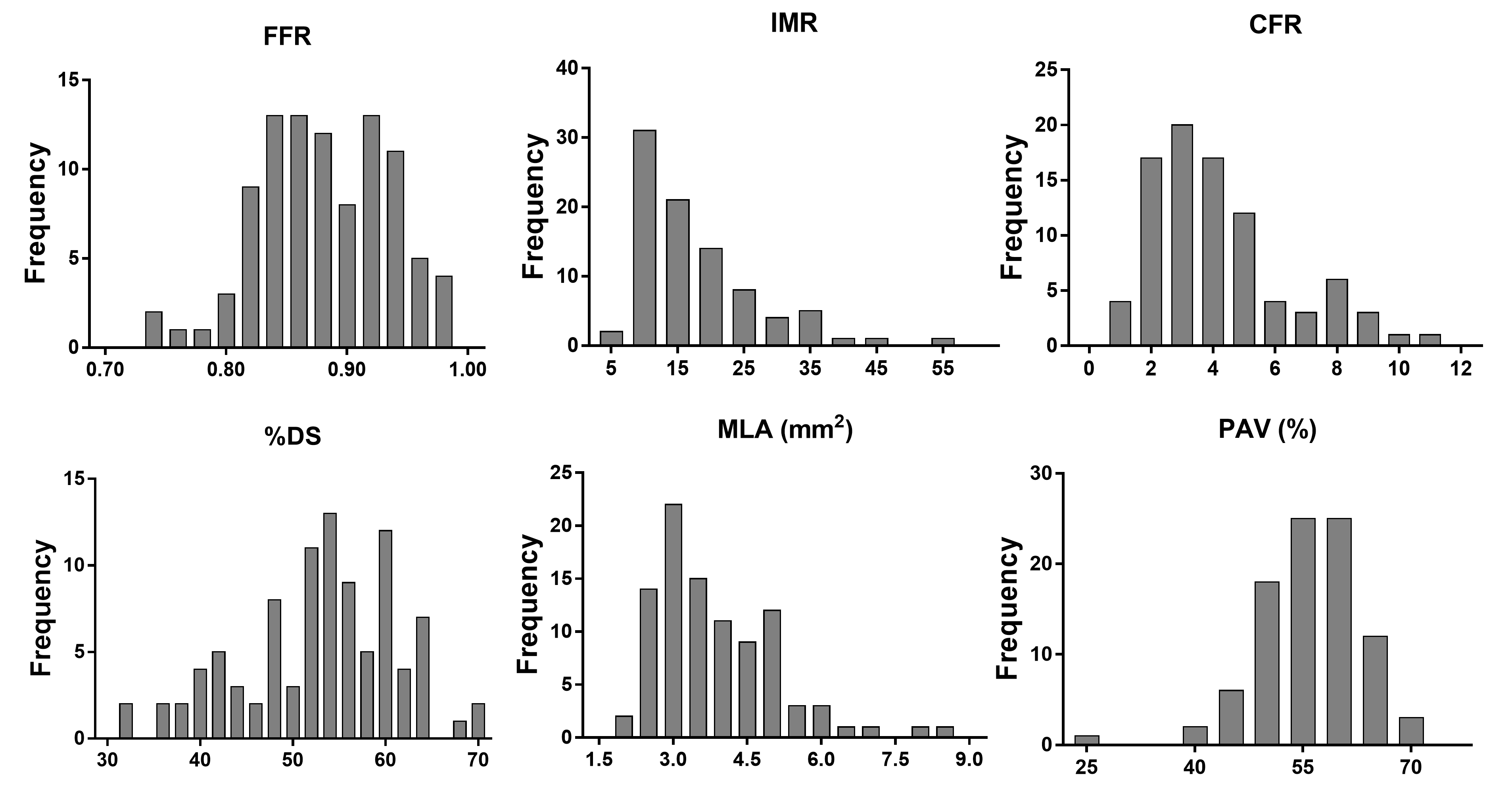
**

**Supplemental Figure 2.** **Changes in Coronary Flow Reserve and Index of Microcirculatory Resistance According to the Dose of Atorvastatin Therapy**


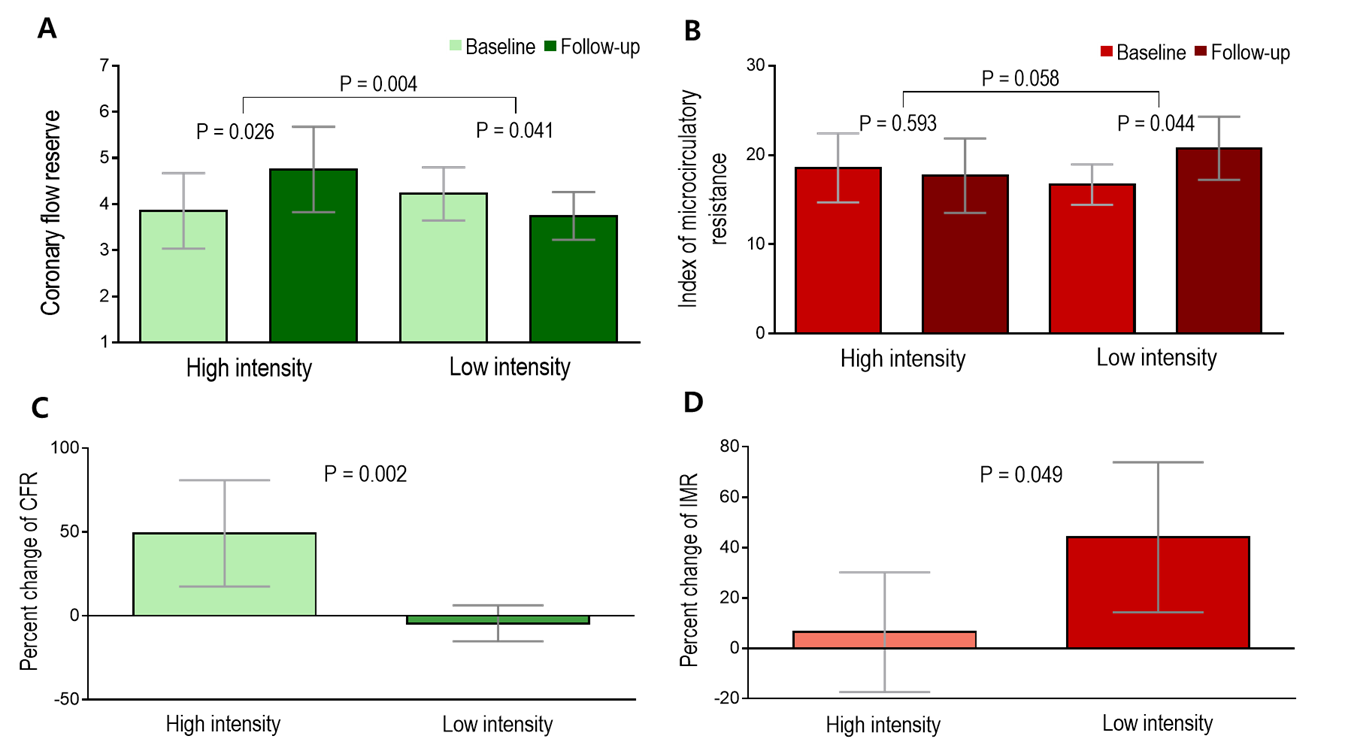

Supplement: Supplemental Figures 1 and 2 and Tables 1–5 [file mmc1.docx]
